# Dyslipidaemia in Type 1 Diabetes: Molecular Mechanisms and Therapeutic Opportunities

**DOI:** 10.3390/biomedicines9070826

**Published:** 2021-07-16

**Authors:** Stephen T. O’Brien, Orla M. Neylon, Timothy O’Brien

**Affiliations:** 1 Department of Paediatrics, University Hospital Limerick, V94 F858 Limerick, Ireland; s.obrien1407@gmail.com (S.T.O.); orla.neylon@hse.ie (O.M.N.); 2 Department of Medicine, School of Medicine, National University of Ireland, H91 TK33 Galway, Ireland

**Keywords:** Type 1 diabetes mellitus, dyslipid(a)emia, atherosclerosis

## Abstract

Cardiovascular disease (CVD) is the leading cause of death in Type 1 Diabetes (T1D). The molecular basis for atherosclerosis in T1D is heavily influenced by hyperglycaemia and its atherogenic effects on LDL. Ongoing research into the distinct pathophysiology of atherosclerosis in T1D offers exciting opportunities for novel approaches to calculate CVD risk in patients with T1D and to manage this risk appropriately. Currently, despite the increased risk of CVD in the T1D population, there are few tools available for estimating the risk of CVD in younger patients. This poses significant challenges for clinicians in selecting which patients might benefit from lipid-lowering therapies over the long term. The current best practice guidance for the management of dyslipidaemia in T1D is generally based on evidence from patients with T2D and the opinion of experts in the field. In this review article, we explore the unique pathophysiology of atherosclerosis in T1D, with a specific focus on hyperglycaemia-induced damage and atherogenic LDL modifications. We also discuss the current clinical situation of managing these patients across paediatric and adult populations, focusing on the difficulties posed by a lack of strong evidence and various barriers to treatment.

## 1. Introduction

CVD is the most common cause of death in Type 1 Diabetes (T1D) [1] and suboptimal glycaemic control is a significant risk factor for CVD [2]. However, even in patients with HbA1c levels lower than the recommended levels of <6.9% (52 mmol/mol), there is still a twofold increase in risk of death and CVD compared with controls [3]. Therefore, factors other than hyperglycaemia must contribute to the accelerated atherosclerosis seen in T1D. One of these other risk factors is dyslipidaemia, with a recent large-scale epidemiological study of patients with T1D showing a 35–50% increase in risk of CVD for every 1 mmol/L increase in LDL-C [3]. Additionally, the presence of multiple risk factors is seen early in the disease process. Population-based studies estimate that two or more risk factors for CVD are present in 15–45% of adolescents with T1DM and increase with age [4,5,6]. Overall, cardiovascular events occur earlier and more commonly in individuals with Type 1 Diabetes [7]. In the UK, a GPRD study found that CVD events occurred an average of 10–15 years earlier than in non-diabetic controls. They reported the hazard ratio for major CVD was 3.6 (95% CI 2.9–4.5) in men and 7.7 (CI 95% 5.5–10.7) in women versus those without diabetes [8]. Recent data have suggested that improved management of cardiovascular risk factors and more intensive glycaemic control have improved the rates of cardiovascular disease in T1D. Analysis of the Swedish nationwide registry data showed significant reductions in the incidence of cardiovascular complications between 1998 and 2014 with a relative event rate reduction of 42% [9]. The recent report from the DCCT/EDIC showed a reduction in all-cause mortality and death secondary to cardiovascular disease in patients assigned to the intensive treatment protocol [10]. However, despite the advances in treatment, T1D is still strongly associated with the development of CVD, with the condition being more strongly linked to CVD outcomes than any other condition in the recent NHS study on the development of the QRISK 3 calculator [11].

The majority of cases of T1D are diagnosed in the paediatric population and the incidence of the condition appears to be increasing [12]. This is important as the morbidity related to atherosclerosis is observed in adult patients, but there is evidence that the onset of atherosclerosis begins in childhood. The Bogalusa heart study demonstrated the presence of fatty streaks and fibrous plaques in the aortas and coronary arteries of young people at post-mortem. The extent of these findings was positively correlated with known CVD risk factors including dyslipidaemia, hypertension, and obesity [13].

The high morbidity and mortality associated with CVD in Type 1 Diabetes suggests a need for early identification of risk factors and evidence-based measures to attenuate that risk. Much of the current guidance on the management of dyslipidaemia in T1D is based on studies composed primarily of patients with T2D despite the differing underlying pathophysiology. There are few clinical trials that have investigated the approach to dyslipidaemia in T1D, in either paediatric or adult cohorts. The aim of this review article is to describe the pathophysiology of dyslipidaemia in T1D and to examine the current evidence for the screening and management of dyslipidaemia in patients with T1D across both paediatric and adult populations.

## 2. Pathophysiology of Atherosclerosis in Type 1 Diabetes

There is evolving evidence that differences exist in the pathophysiology of atherosclerosis in individuals with T1D versus individuals who do not have diabetes. Examination of atherosclerotic plaques in patients with diabetes has shown that they are more lipid-rich and have an increased amount of macrophages and thrombus than those in non-diabetic patients [14]. The pathophysiology of atherosclerosis in T1D is not fully understood. However, early and severe atherosclerosis is a hallmark of T1D. Current hypotheses stipulate that the metabolic abnormalities associated with T1D, i.e., hyperglycaemia, dyslipidaemia, and potential insulin resistance (i.e., in adolescents and those with co-existing obesity), leads to an array of circulating factors culminating in endothelial dysfunction and resulting inflammation, vasoconstriction, and thrombosis [15]. The key pathophysiological processes are outlined below. 

### 2.1. Endothelial Dysfunction

Endothelial cells line the inner surface of all blood vessels and are responsible for controlling the vessel structure and function through production of vascular mediators including nitric oxide, angiotensin 2, endothelin 1, and prostacyclin. Nitric oxide (NO) is a key regulator of many processes including haemostasis, vascular smooth muscle relaxation, inflammation, and thrombosis [16]. Overall, the actions of NO are vasoprotective and are key to the prevention of atherogenesis. Diabetes results in impaired nitric oxide mediated vasodilation [17] through several mechanisms. Endothelial cells are at an increased risk of developing intracellular hyperglycaemia as they are unable to downregulate glucose uptake [18]. Hyperglycaemia has been implicated in cellular damage through four main pathways: activation of protein kinase C isoforms, increased flux through the hexosamine and polyol pathways, and increased formation of advanced glycation end-products (AGEs) [19]. It has been shown that each of these mechanisms result from a single hyperglycaemia-driven process: overproduction of the superoxide anion by the electron transport chain [20,21]. Superoxide over-production leads to breaks in DNA and this DNA damage leads to the activation of poly(ADP-ribose) polymerase (PARP), a function of which is ribosylation and inhibition of glyceraldehyde phosphate dehydrogenase (GADPH) [22]. GADPH inhibition leads to diversion of glucose away from glycolysis down the four alternate pathways discussed above, resulting in cellular damage. AGEs interact with specific receptors on target cells and lead to increased rates of oxidative damage and promote inflammation and coagulation [23]. Activation of protein kinase C beta also increases the generation of reactive oxidative species and upregulation of pro-inflammatory factors. Superoxide anion directly decreases nitric oxide through formation of the peroxynitrite ion which uncouples NO synthase by oxidising its co-factor tetrahydrobiopterin (BH4), causing NOS to produce further reactive oxygen species [24]. 

In addition to reduced nitric oxide bioavailability, diabetes is associated with increased production of vasoconstrictors, the most important of which is endothelin-1. Increased activity of endothelin-1 may be mediated by insulin dependent gene expression, increased gene transcription caused by oxidised LDL or stimulation of the receptor for AGEPs [25,26]. Endothelin-1 increases vascular smooth muscle tone, increases renal salt and water retention activating the renin–angiotensin–aldosterone system, and stimulates vascular smooth muscle hypertrophy [27]. 

The pathways above also result in an increased production of the transcription factors NFKB and activator protein 1 which regulate the expression of many atherosclerotic mediators. This leads to increased expression of leucocyte adhesion molecules, monocyte attracting chemokines, and pro-inflammatory mediators found in atheroma such as TNF alpha and leukotriene-1 [28]. 

Overall, these hyperglycaemia-induced changes lead to significant endothelial damage, resulting in decreased production of nitric oxide and increased endothelin 1/angiotensin 2 with an end result of vasoconstriction and activation of inflammatory mediators. This creates an environment that favours the adhesion of circulating macrophages and invasion into the arterial intima where they are activated and take up lipoproteins, leading to the formation of foam cells and the fatty streak, which are the hallmarks of early atherosclerosis [15,22]. 

### 2.2. Vascular Smooth Muscle Cell Dysfunction

Vascular smooth muscle cells (VSMCs) are key to the development of atherosclerosis. Once fatty streaks are formed, VSMCs migrate to the intima where they lay down a complex extracellular matrix. Hyperglycaemia has the effect of activating protein kinase C, NFkB, and receptors for AGEs in VSMCs as it does in endothelial cells, leading to additional oxidative damage. Enhanced migration of VSMCs has been observed in patients with diabetes. The presence of VSMCs in an atheroma provide collagen and stability, making it less likely to rupture. Plaques which have ruptured and resulted in thrombosis tend to have fewer VSMCs present [29]. However, advanced atherosclerotic plaques in patients with diabetes have fewer VSMCs than in non-diabetic controls [30]. This would suggest that in diabetic atherosclerosis, there is enhanced migration of VSMCs, which aids in progression of the fatty streak to a more advanced plaque, but that there is also increased apoptosis of VSMCs leading to a more unstable atheroma and more clinical events. This may be explained by hyperglycaemic changes to LDL. LDL that has undergone non-enzymatic glycation enhances migration of VSMCs in vitro, while oxidised glycated LDL increases apoptosis of VSMCs [30]. 

### 2.3. Platelet and Coagulation Dysfunction

The concentration of glucose in platelets is equivalent to the extracellular concentration as glucose uptake in platelets is not mediated by insulin [31]. Hyperglycaemia in platelets results in the same pathophysiological process as seen in endothelial cells and VSMCs with resulting production of reactive oxygen species. Hyperglycaemia also appears to confer additional abnormalities in platelet calcium homeostasis and increased expression of surface glycoprotein 1b which may result in an overall decrease in the production of endogenous inhibitors of platelet activity and an increase in intrinsic platelet activity, leading to an enhanced thrombotic potential in these patients [15]. Finally, in diabetes, there is an increased tendency towards coagulation and a decrease in fibrinolysis with an overall increased risk of thrombus formation.

### 2.4. Atherogenic LDL Modifications 

It has been shown that not all LDL particles are atherogenic and that native LDL does not necessarily cause prominent accumulation of lipids in the arterial wall. It is the modification of native LDL that leads to its atherogenic properties. The relationship between small, dense LDL (sdLDL), and dyslipidaemia in diabetes has been extensively researched. The process of LDL modification is thought to be initiated by desialylation of LDL. Sialic acid is an important component of native LDL and it has been proposed that desialylation and multiple subsequent modifications lead to increased atherogenicity [32]. Cholesterol ester transfer protein facilitates the transfer of TGs from triglyceride rich lipoproteins to LDL forming TG-rich LDL which is the preferred substrate of hepatic lipase resulting in increased lipolysis and the formation of sdLDL [33]. Smaller, more dense LDL particles are thought to be more atherogenic for a number of reasons. sdLDL has a longer plasma half-life which increases the probability of an atherogenic modification such as oxidation, desialylation, or glycosylation. The decreased size and density also increase penetration into the arterial wall versus large LDL. sdLDL particles remain in the arterial wall for longer following trans-endothelial transport due to increased interaction with proteoglycans and are more likely to be internalized by intimal cells. Additionally, sdLDL has a lower affinity for binding the LDL receptor; therefore, these particles tend to be internalized through phagocytosis, leading to intracellular accumulation of cholesterol rather than the usual degradation of lipoprotein particles [34]. sdLDL also appears to have lower resistance to oxidative stress due to reduced free cholesterol and altered properties of the surface lipid layer. There is also a decreased antioxidant content, and an increased content of polyunsaturated fatty acids which make sdLDL more prone to oxidation [35]. 

AGEs play a role in multiple steps of atherosclerosis. Glycosylation of LDL particles is a key atherogenic change in the context of chronic hyperglycaemia. Glycosylation occurs on the ApoB and phospholipid portions of LDL and leads to a significant decrease in the recognition of specific LDL by the LDL receptor. This results in decreased LDL-receptor mediated uptake and degradation of glycated-LDL as compared to native LDL. Furthermore, glycated LDL is more readily recognized by macrophages, leading to increased uptake and formation of foam cells via non-specific scavenger receptors on macrophages rather than via the specific LDL-receptor [36]. Glycation of LDL also increases the susceptibility of LDL to oxidation which is considered a crucial step in its atherogenicity. The oxidation of AGE-LDL has been shown to be proportional to the glucose concentration [36]. Aside from the atherogenic effect of AGEs on LDL, they also induce endothelial activation and increased expression of cell adhesion molecules, leading to an increased rate of macrophage migration into the arterial wall and early plaque formation. 

### 2.5. Modified LDL and the Role of the Immune Response and Inflammation in Atherosclerosis in T1D 

In general, inflammation is considered central to the pathophysiology of atherosclerosis and inflammation appears to be more prominent in individuals with diabetes compared with non-diabetic controls. Individual with diabetes have higher levels of inflammatory markers such as interleukin-2, CD-40 ligand, and C-reactive protein [37,38]. The mechanisms by which Type 1 diabetes contributes to a pro-inflammatory state are likely multi-factorial with both hyperglycaemia and hypoglycaemia playing a role. One mechanism of inflammation where a direct link with hyperglycaemia has been made is through a process involving neutrophil extracellular traps (NETs) [39]. Neutrophils release chromatin into the extracellular space in order to “trap” bacteria. This is a mechanism which functions to clear bacteria as the NETs contain bactericidal material and these bacteria are also phagocytosed by macrophages and neutrophils. After releasing NETs, neutrophils die in a process known as NETosis, a recently discovered form of cell death [40]. While this is an effective mechanism for eliminating bacteria, it also serves to induce inflammation through activation of macrophages which release pro-IL-1β, as well as through other mechanisms. There are data that suggest that hyperglycaemia may promote NETosis [41]. The study of anti-inflammatory therapeutics in the management of cardiovascular risk is ongoing, an example of which is canakinumab, a monoclonal antibody against IL-1β currently being examined in diabetes [42].

As discussed, modification of lipids promotes inflammation through increased expression of adhesion molecules and increased release of chemokines and pro-inflammatory cytokines in both macrophages and vascular cells [43]. Above, we discussed the role of LDL modification in the progression of atherosclerosis. It must also be noted that modified LDL particles are immunogenic in nature. Many forms of modified LDL exist and most appear to be immunogenic and induce the formation of auto-antibodies that propagate arterial inflammation [44]. The majority of these antibodies belong to the IgG subclasses (IgG1 and IgG3) [45]. Formation of circulating immune complexes (CICs) between modified LDL and these auto-antibodies is strongly pro-inflammatory, with uptake of these CICs leading to pro-inflammatory activation of macrophages and the release of cytokines such as TNF-α. The study of the use of the levels of mLDL in CICs as a novel biomarker of the risk of CVD in diabetes have shown promising results. It has been shown that total LDL particle levels and levels of modified LDL in CICs are predictive of carotid intima media thickness in T1D [46,47]. The development of assays to measure these parameters represents a potential advance in CVD risk stratification for individuals with T1D. 

## 3. Lipid and Lipoprotein Profiles in Type 1 Diabetes 

In general, individuals with well-controlled Type 1 Diabetes appear to have similar lipid profiles when compared with the general population [48]. However, adverse lipid profiles have been observed in patients with sub-optimally managed diabetes as measured by the glycaemic control and the incidence of complications including nephropathy, hypertension, and obesity [49]. Data in younger people are limited, but the SEARCH study showed similar lipid profiles in adolescents with Type 1 Diabetes with optimised glycaemic control when compared to controls without diabetes. Additionally, the lipid profiles in this cohort (i.e., HbA1c < 7.5%, or 57 mmol/mol) may even be protective against atherogenesis with higher HDL-C, lower triglycerides, and triglyceride:HDL ratios. This “supernormal” lipid profile seen in T1D may be explained by the administration of subcutaneous insulin, which increases lipoprotein lipase activity and subsequently VLDL turnover. In contrast, youths with sub-optimal glycaemic control have higher levels of standard lipids and increased levels of ApoB (a recognised risk factor for CVD) and have smaller, more dense LDL particles than non-diabetic controls [50]. It is worth noting that even with normal standard lipid levels, the underlying lipoprotein composition may still be potentially atherogenic. Youths with optimal levels of HbA1c in the SEARCH study still had higher levels of ApoB and small/dense LDL compared with non-diabetic controls [50]. This correlates well with previous adult studies which have shown similar triglyceride and HDL-C patterns in individuals with more optimised glycaemic control [51]. Another study in adolescents also showed frequent lipid abnormalities, with sustained raised non-HDL-C in 25.9% of patients (mean age at baseline assessment 14.5 years). These abnormalities were shown to be related to increasing age, duration of disease, BMI, and glycaemic control. Additionally, they demonstrated that total cholesterol and non-HDL-C levels were higher in patients with co-existing microalbuminuria (*p* < 0.03), a finding previously demonstrated in adult studies [52]. 

Studies of lipoprotein subclasses in adults with Type 1 Diabetes have shown more conflicting evidence. More advanced analysis of lipoprotein subclasses in individuals with T1D have been published with inconsistent results. Nuclear magnetic resonance spectroscopy is a method which can quantify the number, size, and composition of lipoproteins and can reveal more subtle dyslipoproteinaemia [53]. Further studies published by the DCCT/EDIC have shown a positive association between small and large LDL-P (i.e., the number of LDL particles) and carotid intima media thickness. They also demonstrated an inverse relationship between large HDL-P and CIMT [54]. Two NMR studies have shown proatherogenic patterns in T1D lipoprotein profiles despite having normal standard cholesterol profiles as evidenced by triglyceride rich lipoproteins in adults [53] and high LDL-P with low HDL-P in adolescents [55]. Other studies have suggested a gender difference in lipoprotein subclasses with similar LDL size and subclass between males with T1D and controls, while women with T1D were shown to have more small LDL particles [56]. This may offer an explanation as to why the difference in CVD mortality between male and females seen in the general population is not observed in T1D. In fact, a meta-analysis showed that women with T1D proportionately have a twofold increased risk of fatal and non-fatal CVD events than men with T1D [57]. The DCCT/EDIC study on lipoprotein subclasses showed that the intensive treatment group with a lower HbA1c had a more favourable lipid profile with lower small LDL-P and small HDL-P versus the conventional treatment group [58]. This is in keeping with other studies which have shown an association between glycaemic control and dyslipidaemia [50,59,60]. 

Interestingly, a larger, more recent study of adults with T1D from a Mediterranean population did not replicate the results, showing a more atherogenic profile in T1D. The mean HDL-P size was larger in the T1D group versus healthy controls and the triglyceride content was not found to be increased [60]. Possible factors postulated to have influenced these differences included that this was a newer cohort of patients, possibly reflecting advancements in management of T1D. Additionally, the patients in this cohort are known to be more adherent to the Mediterranean diet, which has been shown to be associated with more favourable lipid profiles [60,61,62]. 

They did, however, note a potentially more atherogenic NMR profile in women. The gender differences seen in the LDL parameters of non-diabetic individuals were not seen between males and females with T1D [60] (i.e., the potential cardioprotective role of female sex was not observed in the T1D group). They also replicated previous results [63], suggesting that insulin resistance is related to VLDL variables and inversely associated with HDL variables. In this study, the markers of insulin resistance used were waist circumference and the FLI fatty liver disease score, and these two markers were the variables most strongly associated with adverse lipoprotein subclasses (in T1D and the controls). They also showed a positive association between glycaemic control and LDL-related variables in their T1D population. Additionally, there was a discrepancy shown between LDL-P and conventional LDL-C measures in the T1D group versus the controls. That is, the frequency of LDL-P >1000 (increased) with an LDL-C <100 (normal range) was higher in the T1D group (38% vs. 21.2%). In the general population, it has been shown that those with a higher LDL-P in relation to conventional LDL-C have a higher risk of future CVD events [64]. This may offer an explanation as to why those with T1D have a higher risk of CVD despite “normal” conventional lipid profiles and may offer a future avenue for risk assessment and patient selection using these more advanced lipid parameters.

## 4. Dyslipidaemia in Type 1 Diabetes: The Clinical Perspective

### 4.1. Current Recommendations and Best Practice Guidelines

#### 4.1.1. Children and Adolescents

The current internationally accepted guidelines regarding lipid management in paediatric Type 1 Diabetes were published in 2018 by the International Society of Paediatric and Adolescent Diabetes (ISPAD) [65].

Screening and Ongoing Surveillance:

- Screening for dyslipidaemia should commence in all children diagnosed with Type 1 Diabetes soon after diagnosis (once diabetes is stabilised) from the age of 11 years.
- If there is a family history of early CVD, hypercholesterolaemia, or if the family history is unknown, screening should begin as early as 2 years of age.
- If the initial screen is normal, this guideline recommends repeat screening every 5 years.
- Fasting lipid profiles are not necessary as the initial screening test. However, if fasting LDL or triglycerides are elevated, fasting studies are then required.

Management:

- High LDL cholesterol is defined as >2.6 mmol/L (100 mg/dL).
- High LDL should be managed initially with lifestyle interventions, including diet and exercise.
- If lifestyle interventions do not lower LDL to ≤3.4 mmol/L, initiation of statin therapy should be considered in children aged >10 years.
- The ideal treatment target is <2.6 mmol/L.

The American Diabetes Association released new guidelines in 2021 entitled Standards of Medical Care in Diabetes. Chapter 13 addresses guidelines for children and adolescents [66]. Their recommendations were similar to those from ISPAD with several subtle differences.

Screening and Ongoing Surveillance:

- Recommend initial lipid screening from 2 years of age.
- If initial screening shows an LDL <2.6 mmol/L, repeat screening at age 9–11 years.
- Repeat screening is recommended every three years if LDL is normal (versus the 5 years recommended by ISPAD).

Management:

- If lipids are abnormal, they recommend initial conservative management with optimisation of blood glucose and medical nutritional therapy to limit the amount of calories from fat to 25–30%, saturated fat to <7%, cholesterol <200 mg/day, avoidance of trans fats, and an aim of approximately 10% of calories from monounsaturated fats.
- Consideration of statin therapy in children over the age of 10 who have persistently raised LDL despite adequate lifestyle interventions and medical nutrition therapy.
- Statins are to be considered with an LDL >4.1 mmol/L or an LDL of >3.4 with one or more additional cardiovascular risk factors.
- The target LDL was the same at <2.6 mmol/L.

#### 4.1.2. Adults

The American Diabetes Association [67] closely reflects the recommendations of the American Heart Association.

Screening and Ongoing Surveillance:

- In adults not taking statins or other lipid-lowering therapy, it is reasonable to obtain a lipid profile at the time of diabetes diagnosis, at an initial medical evaluation, and every 5 years thereafter if under the age of 40 years, or more frequently if indicated.

Management:

- Lifestyle interventions focusing on weight loss (if indicated); application of a Mediterranean style or Dietary Approaches to Stop Hypertension (DASH) eating pattern; reduction of saturated fat and trans fat; increase in dietary n-3 fatty acids, viscous fibre, and plant stanols/sterols intake; and increased physical activity should be recommended to improve the lipid profile and reduce the risk of developing atherosclerotic cardiovascular disease in patients with diabetes.
- Recommend a lipid profile at initiation of statins or other lipid-lowering therapy, 4–12 weeks after initiation or a change in dose, and annually thereafter as it may help to monitor the response to therapy and inform medication adherence.

Pharmacotherapy for Primary Prevention:

- 40–75 years: Moderate intensity statin therapy for all patients with diabetes without ASCVD. Additionally, if there are multiple ASCVD risk factors present in a patient between 50–70 years, it is reasonable to use high-intensity statin therapy.
- In adults with a 10-year ASCVD risk >20%, it may be reasonable to add ezetimibe to maximally tolerated statin therapy with an aim to reduce LDL-C >50%.
- The recommendations for patients with diabetes less than 40 years of age and/or T1D with other CVD risk factors is not entirely clear as the evidence in this group is lacking.
- They state that similar statin treatment approaches should be considered for patients with Type 1 and Type 2 diabetes, particularly in the setting of additional risk factors. They recommend that the health care provider has a discussion regarding the relative risks and benefits and to consider the use of moderate intensity statin therapy.

Pharmacotherapy for Secondary Prevention:

- All patients with diabetes and atherosclerotic cardiovascular disease should be commenced on high-intensity statin therapy in addition to lifestyle interventions.
- In patients with very-high risk ASCVD with an LDL >1.8, consider the addition of a non-statin cholesterol-lowering medication such as a PCSK9 inhibitor or ezetimibe.

European Guidelines:

EAS/ESC [68]

- Statins are recommended in all patients with T1D who are in the “high risk or very high-risk category”.
- High Risk: Patients with DM without target organ damage, with DM duration >10 years, or another additional risk factor.
- Very High Risk: DM with target organ damage, or at least three major risk factors, or early onset of T1D of long duration (>20 years).
- Statin therapy may be considered in both T1D and T2D patients aged <30 years with evidence of end organ damage and/or an LDL-C >2.5 mmol/L, as long as pregnancy is not being planned.

NICE guidelines on lipid modification from 2014 [69]

- Recommend offering atorvastatin 20 mg as primary prevention of CVD to people with T1D who are >40 years old, have had diabetes for more than 10 years, have established nephropathy, or have other CVD risk factors.

## 5. CVD Risk Assessment and Calculators 

All current guidelines for the management of dyslipidaemia in general recommend the calculation of total cardiovascular risk. There are many CVD risk calculators in use—the ESC/EAS guidelines recommend using risk charts based on country-based cohort data where possible. The SCORE (Systematic Coronary Risk Estimation) can be recalibrated for use in different populations and is the calculator recommended by the ESC [68]. The ADA recommend the American College of Cardiology/American Heart Association ASCVD risk calculator, which can be used to estimate the ten-year risk of a first ASCVD event [67]. This calculator includes diabetes itself as a risk factor but does not consider duration of disease or the presence of complications such as nephropathy or retinopathy and therefore may underestimate risk in T1D. One clinical risk score has been developed specifically for use in patients with T1D with no prior CVD event [70]. This is a promising step towards risk stratification in T1D but needs further validation. A recent Italian study showed that this calculator identified patients with subclinical atherosclerosis and high-risk patients but overestimated the absolute risk of ASCVD events [71]. Scores from this calculator have also been highly correlated with arterial thickness, another potential promising use for detecting subclinical atherosclerosis [72]. Finally, an updated version of the QRISK 10-year calculator was published in 2017 entitled QRISK3 [11]. Type 1 Diabetes was included as a separate variable in this iteration (versus QRISK2). NICE guidelines in 2014 recommended the use of QRISK2 in Type 2 diabetes but not in T1D. The authors of the recent paper on QRISK3 assert that it should be used in the calculation of risk in T1D to better facilitate discussions on the risks and benefits of lipid-lowering medications in this group. The performance of these models was actually higher in patients with T1D versus T2D [11]. The use of long-term risk calculators for T1D is not commonplace in clinical practice in paediatrics. However, as discussed above, the onset of atherosclerosis has been shown to commence in childhood and children with T1D are at a particularly high risk of long-term CVD complications. 

Screening for dyslipidaemia is currently carried out clinically through assessing the standard lipid profile. There is ongoing research into the use of novel cardiac biomarkers to build individual risk profiles for patients. These biomarkers could potentially be incorporated into T1D-specific risk calculators to increase their accuracy. Candidate biomarkers that have been shown to correlate with CVD include ApoB, lipoprotein A, modified LDL (e.g., oxidised LDL) and LDL-immune complexes, receptors for AGEs, metalloproteinases, and highly sensitive C-reactive protein [73]. Currently, the cost of testing for these markers prohibits their use in clinical practice and they are mainly used in the research setting. Development of commercially available and reliable assays for testing these biomarkers would be key in their introduction into clinical risk calculators. Among the most readily available and important tools for cardiac screening in the clinical setting is the electrocardiogram. During follow-up, the development of ECG changes is common in T1D, with one study showing at least one new ECG abnormality in 77.3% (major or minor) of patients over a 16-year follow-up. Furthermore, 13.1% had a major ECG change during the follow-up period [74]. This suggests regular ECGs are useful in monitoring CVD in T1D. 

Lastly, there is potential for the incorporation of imaging modalities into risk calculators in T1D. A recent prospective study of the use of echocardiography for the assessment of CVD risk in individuals with T1D without cardiovascular disease showed that measures of myocardial dysfunction are significantly associated with major adverse cardiovascular events over a 7-year follow-up. These echocardiographic parameters were shown to identify at-risk individuals at an improved rate compared with conventional risk factors alone [75]. Carotid ultrasound can be used to assess cIMT (a good surrogate marker of atherosclerosis) which is increased in T1D and is a predictor of CVD events [73]. There is also increasing use of computed tomography coronary angiography (CTCA) for identifying atherosclerosis. CTCA can be used to generate coronary artery calcification (CAC) scores. CAC is increased in T1D versus the general population and is associated with CVD. Additionally, improved glycaemic control has been shown to reduce progression of CAC scores in T1D [48]. 

Overall, patient selection criteria for the treatment of dyslipidaemia in T1D in younger patients is unclear due to the lack of evidence. Development of accurate risk calculators for this population would be of great benefit going forward.

## 6. Current Evidence for Management

### 6.1. Non-Pharmacological 

#### 6.1.1. Paediatrics 

The initial management of dyslipidaemia in children as recommended by best practice guidelines revolves around lifestyle interventions including diet and exercise. A 6-month-long Italian trial showed that a dietician-led programme prioritising a Mediterranean-style diet improved the quality of nutrient intake and subsequently showed a decrease in LDL-C and non-HDL-C [76]. Another 6-month trial evaluating the effect of a supervised exercise programme showed improvements in dyslipidaemia and a decrease in insulin requirements [77]. It has also been shown that cardiorespiratory fitness is inversely correlated with non-HDL-C, TG, and TC in children and adolescents with T1D [78].

In addition to these lifestyle interventions, improved glycaemic control has been shown to improve lipid profiles, but may not be sufficient to fully normalise dyslipidaemia alone [79]. Intensive insulin regimens have been shown to improve endothelial function in young people with T1D and this benefit was shown to be independent of changes in HbA1c [80].

#### 6.1.2. Adults 

Similar to paediatric guidelines, adult diabetes guidelines recommend lifestyle interventions for the management of dyslipidaemia. The Mediterranean diet when supplemented with extra virgin olive oil or nuts was associated with a lower rate of major adverse cardiovascular events [81]. This diet consists of a high intake of olive oil, nuts, fruits, and vegetables; a moderate intake of fish and poultry; and a low intake of red meat, sweets, and dairy. Additional dietary recommendations include the DASH (Dietary Approaches to Stop Hypertension) programme. This diet has shown to effectively reduce blood pressure and is regularly recommended in cardiovascular guidelines. However, adherence to DASH was shown to be very low in the USA and innovative population-based interventions are required to improve engagement with these programmes [82].

### 6.2. Pharmacological 

#### 6.2.1. Paediatrics 

Current recommendations on management of dyslipidaemia in paediatrics are based on adult studies or on evidence of preclinical atherosclerosis, as there are no trials in paediatrics showing a relationship between treatment cut-offs and a decrease in CVD events in the future. Effective lifestyle interventions to change behaviour can be exceedingly difficult to achieve and may be insufficient on their own for optimal cardiovascular health [83,84]. Furthermore, it has been well established that cardiovascular risk factors in this patient population are undertreated [85]. A recent study of dyslipidaemia in children with T1D showed that of those who met the criteria for statin prescription, only 42% had one prescribed [86]. Most of the data on the safety and efficacy of statins in children and adolescents are derived from studies on familial hypercholesterolaemia. Systematic reviews on the use of statins in FH have established that they are efficacious and safe for use, though longer-term studies are required to assess lifelong safety [87]. Early intervention with statins in FH has improved endothelial function and caused regression of carotid IMT [88,89]. Few trials have been carried out of statin use in paediatric T1D. The PADIT trial did show that atorvastatin significantly lowers LDL-C in children with Type 1 Diabetes and has an excellent short-term safety profile [90]. The ADDIT trial examined the use of ACE inhibitors and atorvastatin in children over 2–4 years. This confirmed the safety of atorvastatin in this age group. Statins also resulted in significant reductions in total cholesterol, LDL, TG, and ApoB:Apo1a ratios [91]. Statin use is not approved in children <10 years of age and should not be used in this age group (an exception is familial hypercholesterolaemia, where treatment is recommended to begin from diagnosis in homozygous patients). Suggested barriers to treatment include the risk of teratogenicity associated with statin use. Statins are currently contraindicated in pregnancy, and the ADA guidelines recommend avoiding statins in women of child-bearing age who are sexually active and not taking appropriate contraception. It is known that cholesterol is important for foetal development and therefore statins which interfere with cholesterol production could have teratogenic potential. However, the evidence for the teratogenicity of statin use comes primarily from animal studies. Previous systematic review of statin use in animal studies show conflicting results. Animal studies that did demonstrate teratogenicity used higher doses of statins than commonly used in clinical practice and significant toxicity was noted in the animal mother at these doses [92]. Interestingly, a recent systematic review of the use of statins in pregnancy did not find any clear relationship between statin use and congenital anomalies and the study found that statins were unlikely to be teratogenic. It included 16 human studies (and excluded animal studies), of which 5 were case-series (n = 755 subjects), 3 were cohort studies (n = 1465 subjects), 3 were registry-based studies (n = 131 subjects), 4 were systematic reviews, and 1 was a randomised controlled trial of pravastatin use in pre-eclampsia (n = 20 subjects). The available evidence was not sufficient to conclusively ascertain whether statins are safe in pregnancy as the data are sparse and they concluded that until more information was gathered, statins should be avoided in pregnancy [93]. Additional barriers may include the lack of long-term efficacy data and patient/parental opposition to additional medication use.

#### 6.2.2. Adults 

There is evidence that LDL-C is among the most important risk factors for myocardial infarction (along with duration of disease and HbA1C) in T1D with each 1 mmol/L increase in LDL-C associated with a 35–50% increased risk of ASCVD events. The study showed that LDL-C has an almost linear relationship with the risk of outcomes, especially death and myocardial infarction. Additionally, there is evidence to suggest that the optimum levels of LDL-C for reducing risk of cardiovascular disease may be lower than currently recommended by guidelines [3]. 

The strongest evidence for statin use in diabetes in general exists in the 40–75-year-old group, with several randomised controlled trials of moderate intensity statin use leading to reductions in ASCVD (these trials mainly consisted of individuals with T2D) [94,95,96]. However, little evidence exists for the management of dyslipidaemia in T1D at any age. Regardless, the AHA guidelines suggest treating all individuals with T1D with moderate intensity statin therapy in the 40–75-year group based on the available evidence [97]. The heart protection study included a sub-group of approximately 600 patients with Type 1 DM with a lower age limit of 40 years. They showed a similar decrease in risk as the T2D cohort; however, it was not found to be statistically significant [96]. A meta-analysis of 18,686 patients (which included the heart protection study) consisted of 1466 patients with T1D and 17,720 with T2D. This showed a relative risk reduction in ASCVD events of 22% with statin therapy. However, again this was not found to be statistically significant although no difference was found compared to T2D patients [98]. A meta-analysis examined the use of high-intensity statin therapy and guidelines have subsequently suggested their use in patients with diabetes between 50–70 years with additional CVD risk factors [97,99]. However, it should be noted that no clinical trials of high-intensity statin therapy exclusively in patients with diabetes have been carried out.

The evidence for lipid-lowering medications in T1D in patients <40 years is lacking and the age at which statin therapy should be commenced is largely unclear. While the short-term risk of ASCVD in this age group is generally low, there is evidence that the risk increases with time, especially in those with a long duration of disease (>20 years) [100]. Children and teenagers who are diagnosed at a young age with diabetes have significant rates of complications including nephropathy, neuropathy, and retinopathy in late adolescence and early adulthood. The risk is more pronounced in T2D but is still significant in patients with T1D [101]. The ESC/EAS guidelines use risk categories to determine when to commence statins in this age group and recommend commencing a statin in all T1D patients who are “high or very high risk”. The main discriminators of risk are duration of disease >10 years, target organ damage (particularly microalbuminuria), and a number of cardiovascular risk factors [68]. Many of the recommendations for commencing statins in this age group are based on evidence that established complications are associated with an increased risk of cardiovascular disease [102,103] rather than clinical trial evidence showing their efficacy in preventing ASCVD events long term.

## 7. Discussion and Recommendations

Despite the well-documented increased risk of CVD in T1D and the established role dyslipidaemia plays in atherosclerosis, a significant amount of uncertainty still exists regarding optimal management strategies for these patients. As discussed, there is strong evidence that atherosclerosis begins in childhood and that children with early onset diabetes have high rates of complications in early adulthood. Many of the recommendations made in paediatric guidelines are based on data from adult trials (where the evidence is also sparse) and rely on expert consensus or opinion. Nevertheless, these guidelines do give clear indications for statin use and clinical targets. In practice, however, we have seen that management of this patient group is not reflective of published guidelines. The barriers to treatment in paediatrics include persistent concerns for the teratogenic potential of statins and consequently their use in adolescent females. Additionally, young people and their parents may be reluctant to commence further medications when already contending with the heavy burden of multiple insulin injections. Parental education is made more difficult by the fact that there is no long-term safety or efficacy evidence to point to in the discussion for commencing statins. What are the main areas that need to be addressed to enhance treatment in this group? Further research into the lipoprotein subclasses in youths with T1D and their association with the development of atherosclerosis could be useful in the assessment of risk and selection of patients for treatment. More information on the teratogenicity of statins would be significant. Most importantly, RCTs examining the efficacy and safety of statin use exclusively in younger people with T1D that includes long-term follow-up would mark a significant advance in clinical practice.

At a basic level, ongoing research into the differing pathophysiology of atherosclerosis in T1D vs. the general population and even T2D could yield promising treatment targets. More research is required to evaluate the role of lipoprotein subclasses in risk stratification of patients with T1D, as the results so far have been conflicting. There were also discrepancies seen between results in adult patients versus those in children and adolescents. Possible explanations for these discrepancies are the effects of pubertal hormones on insulin resistance, which could affect lipoprotein subclasses. The higher insulin requirements seen in adolescence could also play a role. Additionally, use of lipid-modifying drugs could account for some of the differences seen, with much lower frequency of statin use reported in the paediatric studies than those in adults. 

The issues regarding selecting ideal candidates for statin therapy and the optimal age at which to commence treatment is also present in adult practice. There is a similar lack of randomised controlled trial evidence for the use of statins in T1D, especially under the age of 40 years. Key questions that need to be answered include what is the significance of CVD risk factors in adolescents and young adults and what are the indications to intervene? When is the optimal time to commence statin therapy, and in whom? Are statins safe and efficacious over the long term in this population?

Tools for risk-assessment and therefore correct patient selection for treatment will be essential going forward. The further validation of the existing CVD risk calculators for T1D and the development of novel calculators could play a key role in patient selection. Further research into novel biomarkers of CVD in T1D is another exciting research opportunity for risk stratifying younger people with T1D. A promising area is the evaluation of the role of modified LDL (oxidised LDL, AGE-LDL, and malondialdehyde-LDL), which are immunogenic and result in the production of autoantibodies. These antibodies then form circulating immune complexes with modified LDL and these LDL-immune complexes (LDL-ICs) have been shown to be pro-inflammatory and pro-atherosclerotic. There is accumulating evidence that quantifying modified LDL and associated immune complexes may have a better predictive value than conventional lipid measurements [104]. Measurement of LDL-immune complexes at baseline (i.e., years before the onset of any clinical atherosclerotic disease) was associated with CVD events over a 25-year period even after adjusting for other risk factors including LDL-C [105]. This suggests that measurement of LDL-IC early in the disease process could be used to highlight high-risk individuals many years before clinical atherosclerosis. Commercial development of assays that detect oxLDL would need to be developed and validated for widespread use. Those patients identified as being high risk could be treated early and aggressively. Another area worthy of consideration is the use of newer imaging modalities which may be used to detect subclinical disease with the potential to target therapies to those most likely to benefit. 

In summary, the risk of CVD is well documented in this group. The difficulty remains in the selection of the appropriate younger patients who may benefit from lipid-lowering drugs, the treatment of whom will be long-term by virtue of patient age. The main advances that need to occur to overcome this issue are RCT evidence for the longer-term safety and efficacy of statins in T1D specifically. Additionally, new approaches to individually assess risk in T1D patients would significantly improve and standardise management of risk factors in T1D. As discussed, keys to this include further evaluation and optimisation of risk calculators and the discovery and development of novel biomarkers and imaging methodologies in order to create individualised risk profiles going forward. This will represent a personalized medicine approach to management.

## 8. Conclusions

Cardiovascular disease remains the leading cause of death in T1D despite advances in glycaemic control and risk factor management. Ongoing research into the pathophysiology of atherosclerosis and the lipoprotein subclasses in T1D is essential to our understanding of the disease and may offer new targets for therapy. What remains clear is that there is a significant gap in evidence from randomised controlled trials for the management of dyslipidaemia in both paediatric and adult individuals with T1D. Significant advances have been made in the management of this condition, but more clinical research in this area is required to guide clinicians and to inform evidence-based practice.

## Data Availability

Not applicable.
